# Experimental Investigation of Fatigue Crack Growth Behavior of the 2.25Cr1Mo0.25V Steel Welded Joint Used in Hydrogenation Reactors

**DOI:** 10.3390/ma14051159

**Published:** 2021-03-01

**Authors:** Yan Song, Mengyu Chai, Zelin Han

**Affiliations:** School of Chemical Engineering and Technology, Xi’an Jiaotong University, Xi’an 710049, China; songyan1211@mail.xjtu.edu.cn (Y.S.); hanzelin@stu.xjtu.edu.cn (Z.H.)

**Keywords:** fatigue crack growth, welded joint, acoustic emission, 2.25Cr1Mo0.25V steel

## Abstract

In this work, the fatigue crack growth (FCG) behavior and fatigue damage mechanism of the 2.25Cr1Mo0.25V steel welded joint used in hydrogenation reactors were investigated. The multi-pass welding was carried out to manufacture the welded joint using the combined shielded metal arc welding (SMAW) and submerged automatic arc welding (SAAW) processes. The FCG behavior of different zones in the welded joint, including the base metal (BM), the heat-affected zone (HAZ) and the weld metal (WM), were studied by compact tension tests. Moreover, the acoustic emission (AE) technique was used to monitor AE signals generated from FCG process for further understanding FCG behavior and fatigue mechanisms. Additionally, the microstructures and fracture surfaces of different specimens were observed by optical microscopy (OM) and scanning electron microscopy (SEM). The results revealed that the microstructure of BM is fine granular bainite, while the WM shows coarser bainite grains. The HAZ exhibits the most significant inhomogeneity with large dispersion of grain size. FCG results showed that the HAZ exhibits much higher fatigue crack growth rate (FCGR) at low Δ*K* values, while the BM shows the most superior fatigue resistance. The AE technique is successful in monitoring and identifying damage evolutions during the FCG process. Moreover, an enhanced AE activity is observed in FCG of the WM specimen, which is attributed to the combined influence of the formation of numerous secondary cracks and coarse-grained microstructures.

## 1. Introduction

Hydrogenation reactor plays a crucial role in petrochemical hydroreforming, hydrorefining and hydrocracking industries. The equipment is generally operated in harsh service environment, such as high temperature and high pressure conditions. The materials that possess high mechanical properties and excellent resistance to corrosion and hydrogen damage are; therefore, needed. However, the traditional high strength low alloy Cr-Mo steels, for example, 2.25Cr1Mo steel, are not suitable to be applied in the extreme service conditions due to their relatively low strength and poor performance of resistance to hydrogen damage [1]. Therefore, a new vanadium (V)-modified Cr-Mo steel (i.e., 2.25Cr1Mo0.25V steel) with superior mechanical properties has been developed and is being employed in the fabrication of hydrogenation reactors [1,2].

In general, hydrogenation reactors suffer severe cyclic loads, such as numerous startup-shutdown operating cycles during their entire service history. The cyclic stress will inevitably cause considerable stress concentration in the defects of the material, for instance, the inclusions in welded joint, leading to fatigue crack initiation. The further propagation of fatigue cracks will cause serious failure of equipment once the crack size exceeds the critical size. In recent years, a large number of studies focused on the microstructures and mechanical properties [3,4,5], reheat cracking sensitivity [6,7], creep behavior [8] and hydrogen-induced damage [4,9] of 2.25Cr1Mo0.25V steel; however, the study of fatigue crack growth (FCG) behavior is limited. For instance, Peral et al. [10] investigated the combined effects of the pre-charged hydrogen and loading frequency on FCG behavior of 2.25Cr1Mo and 2.25Cr1Mo0.25V steels. The results showed that the presence of internal hydrogen caused an important increase in the crack propagation rate, and this effect increased with the decrease of test frequency. Zhao et al. [11] investigated the low cycle fatigue behavior of 2.25Cr1Mo0.25V steel at 728 K in air by using different strain amplitudes. The results showed that the steel failed from propagation of transgranular fatigue cracks. Moreover, the cyclic softening was observed during whole fatigue life, and such behavior can be accelerated by the increase of strain amplitude. The limited work on fatigue behavior of 2.25Cr1Mo0.25V steel greatly hinders the solid understanding of the fatigue fracture mechanism as well as the fatigue life prediction.

On the other hand, welding is commonly employed in the fabrication of hydrogenation reactors. The welded joint is generally considered as one of the most critical parts in equipment, and can be commonly divided into three regions (i.e., the base metal (BM); the heat affected zone (HAZ), which is located just beneath the fusion line; and the weld metal (WM)). In particular, the HAZ and WM are likely to become the weak parts under stress due to microstructural inhomogeneity caused by complex thermal history. For example, it has been reported by Tsay et al. [12] that the fatigue crack growth rate (FCGR) of HAZ was higher than that of other regions in the 2.25Cr1Mo steel welded joint. Deng et al. [13] investigated the FCG behavior of the dissimilar welded joint made from advanced 9Cr and 2.25Cr1Mo0.25V steels. The results showed that the 9Cr-HAZ with narrow size was the weaker part with higher crack growth rate, compared with other parts of the whole welded joint. Our previous work also demonstrated that the WM showed poorer performance of fracture toughness and resistance to hydrogen embrittlement than those of BM in the 2.25Cr1Mo0.25V steel welded joint [14,15]. Fully understanding the fatigue behaviors and damage mechanisms of different zones (BM, HAZ and WM) of welded joints is; thus, of great importance in the safety and reliability of hydrogenation reactors. However, to the best of our knowledge, the FCG behaviors and failure mechanisms of different zones in 2.25Cr1Mo0.25V steel welded joint have not been investigated before. 

The present work aimed to investigate the FCG behavior and fatigue mechanism of the 2.25Cr1Mo0.25V steel welded joint. The multi-pass welding was carried out on the steel plates using the combined shielded metal arc welding (SMAW) and submerged automatic arc welding (SAAW) processes. Compact tension (CT) specimens with notches in different regions (i.e., BM, HAZ and WM) were machined from the welded joint. The fatigue tests were performed on an Instron testing machine and the crack growth rates were measured. Moreover, the acoustic emission technique (AET) was used to monitor acoustic emission (AE) signals generated from FCG of different regions. AET is a significant nondestructive testing method, and has been widely utilized in detecting and evaluating crack growth in various materials due to its high sensitivity to microstructural change and excellent capability of online monitoring [16,17]. Previous work has taken advantage of AE to investigate the effects of microstructures such as grain size, phase condition and inclusions on mechanical behaviors of materials [18,19,20,21]. Therefore, in this study, AE signals during FCG were measured to investigate crack growth behaviors and corresponding failure mechanisms of BM, HAZ and WM in the 2.25Cr1Mo0.25V steel welded joint. In addition, the microstructures and fracture morphology of specimens were investigated by optical microscopy (OM) and scanning electron microscopy (SEM), respectively, to clarify the fatigue mechanisms.

## 2. Materials and Methods

**Figure 1:**
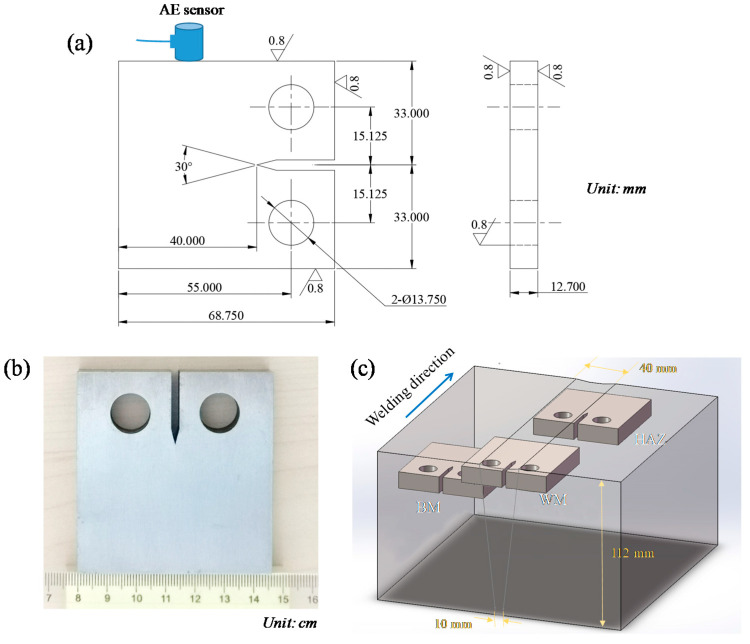
(**a**) Dimensions of the compact tension (CT) specimen. All units are in mm. (**b**) Macrographic photo of the CT specimen. The unit is in cm. (**c**) Schematic of positions of the CT specimens’ sampling.

The 2.25Cr1Mo0.25V steel plates, with a thickness of 112, a width of 250 mm and a length of 800 mm was used for welding in this study. The mechanical properties of 2.25Cr1Mo0.25V steel from tensile test is provided in Table 1. The steel plates were welded by the multi-pass welding method, which was conducted by Lanzhou LS Heavy Equipment Co., Ltd (Lanzhou, China). In particular, the shielded metal arc welding (SMAW) was first applied for root welding, and the remaining passes were performed by submerged automatic arc welding (SAAW). During the SAAW process, the current, voltage and travel speed were maintained at 580 A, 32 V and 316 mm/min, respectively. The choice of these welding parameters were based on the actual manufacture procedure of hydrogen reactors made of CrMo steels. More relevant information for welding can be found in our previous studies [22,23]. After welding, the welded joints were analyzed using X-radiography (XLBG-350TX-5, Xinli, Dandong, China) for locating any defects. The chemical composition of BM and WM of the 2.25Cr1Mo0.25V steel welded joint was measured by spark atomic emission spectrometry (Spark-AES, S600, Boyue Instrument, Nanjing, China) according to ASTM E415 standard [24], and the result is provided in Table 2. The BM, HAZ and WM compact tension (CT) specimens, with a width of 62.5 mm and a thickness of 12.7 mm (see Figure 1a), were machined from the as-welded material according to ASTM E647 standard [25]. The macrographic photo of the CT specimen is shown in Figure 1b. The CT specimens were machined near the top surface of the welded joint and along the welding direction. The sharp notches were located in BM, HAZ and WM to facilitate crack initiation and propagation, respectively, as shown in Figure 1c.

The metallographic specimens (10 × 10 × 10 mm), in different zones extracted from the top surface of the welded joint, were also machined to examine the microstructures of BM, HAZ and WM by using an optical microscope (ECLIPSE, Melville, LA, USA). The preparation of metallographic specimens was conducted based on the ASTM E3 standard [26]. Specifically, these specimens were gradually ground with grinding papers up to 1500 grit and, subsequently, mirror-polished using a 1 μm diamond paste. Then, the specimens were cleaned with de-ionized water and ethanol, and dried with the aid of a stream of cold air. To observe the microstructures, the specimens were etched using a 5% Nital solution, and finally cleaned with ethanol and dried with the help of cold air. In addition, the measurement of grain size was carried out based on the ASTM E112 standard [27].

Fatigue crack growth (FCG) tests were performed according to ASTM E647 standard [25] on an Instron servo-hydraulic testing machine (Instron, Norwood, MA, USA) at room temperature. The sinusoidal cyclic load with a peak load of 20 kN and stress ratio (the ratio of the minimum peak stress to the maximum peak stress of one loading cycle) of 0.1 was applied to the specimens. The test frequency was maintained at 15 Hz for all specimens. To accurately measure the crack size, the direct current potential drop (DCPD) method was used due to its high resolution and excellent stability on crack length increment detection. In particular, a constant current of 2 A was applied through the specimen and the potential difference across the crack was measured by using a nano-voltmeter during fatigue test. The measured crack voltages were then related to the crack lengths. After tests, monotonic uniaxial tensile processes were further carried out on the tested specimens to complete fracture. The fracture surfaces were investigated for understanding fatigue mechanisms using a scanning electron microscope (SEM, SU 3500; Hitachi, Tokyo, Japan).

The AE signals generated during FCG were recorded by using an AE monitoring system (SAMOS, Physical Acoustic Corporation, PAC, Princeton Jct, NJ, USA) to investigate the fatigue mechanisms of different zones in 2.25Cr1Mo0.25V steel welded joint. To collect signals, an AE sensor (R15α) with high sensitivity was mounted on the surface of specimens with the aid of vacuum grease and insulating tape, as shown in Figure 1. A preamplifier with a gain of 40 dB was also used to amplify the AE signals. During FCG tests, an amplitude threshold of 55 dB and a compatible filter of 100-400 kHz were maintained to eliminate the mechanical and environmental noises. Different characteristic parameters such as amplitude, count and energy were extracted from AE waveforms. The definitions of these parameters can be found in [28].

## 3. Results and Discussion

### 3.1. Microstructures

**Figure 2:**
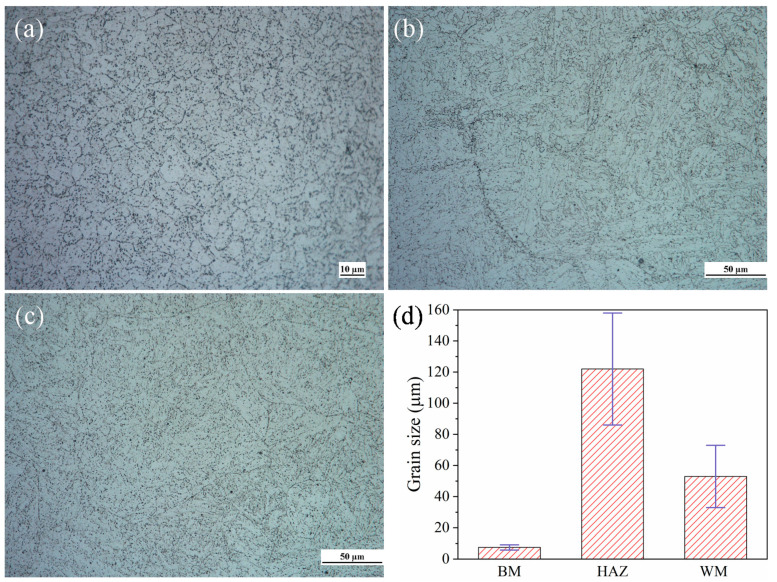
Microstructures of the 2.25Cr1Mo0.25V steel welded joint for (**a**) base metal (BM), (**b**) heat affected zone (HAZ) and (**c**) weld metal (WM), and (**d**) grain size distribution of different zones. Note that the scale bar in (**a**) BM is 10 μm, while in (**b**) HAZ and (**c**) WM is 50 μm. The error bars in (**d**) represent the standard deviation.

Figure 1 shows the typical microstructures of BM, HAZ and WM in the 2.25Cr1Mo0.25V steel welded joint. It is obvious that the main microstructure of BM is fine granular bainite, while WM exhibits coarse bainite grains, whose grain size are much larger than BM. Moreover, the microstructure of HAZ exhibits the lath-like bainite morphology with coarse grains (see Figure 2b). It is noteworthy that the grain size in HAZ is much larger than that in other regions of the welded joint. During welding, the HAZ, located next to the fusion line, attains a very high peak temperature thermal cycle, which allows the previously precipitated carbides to enter into solution, promoting a significant grain growth [29,30]. As a result, after cooling, the HAZ is characterized by a coarse-grained microstructure. The grain sizes were determined by ImageJ software (National Institutes of Health, NIH, Bethesda, MD, USA) using at least 10 metallographic figures obtained on different areas of each specimen. Figure 2d shows the grain size distribution results. The average grain sizes of the BM, HAZ and WM were obtained as 7.5, 121 and 53 μm, respectively. Moreover, the microstructures of HAZ and WM exhibit significant inhomogeneity due to large dispersion of their grain sizes. Thus, different FCG and AE behaviors are expected for the three regions in the welded joint.

### 3.2. FCG Behavior

**Figure 3:**
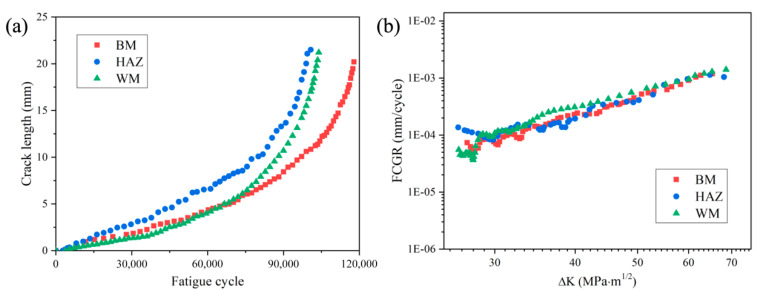
(**a**) Fatigue crack length versus fatigue cycles. (**b**) Fatigue crack growth rate versus the stress intensity factor range.

The stress ratio of 0.1 and the frequency of 15 Hz were used to test the FCG behaviors of different zones in the welded joint. The relationship of fatigue crack length with fatigue cycles is shown in Figure 3a. It is obvious that the fatigue crack propagates more rapidly in HAZ specimen, whereas BM shows the most superior fatigue resistance with the longest fatigue life. The fatigue crack growth rates (FCGRs) of different specimens were calculated by using the seven-point incremental polynomial method according to ASTM standard E647 [25]. The relationship of FCGR to the stress intensity factor range (Δ*K*) is displayed in Figure 3b. In particular, the stress intensity factor range indicates the crack driving force, and the Δ*K* of CT specimen can be calculated according to the following equation [25]:

(1) $$\Delta K=\frac{\Delta P}{B\sqrt{W}}\frac{\left(2+\alpha \right)}{{\left(1-\alpha \right)}^{3/2}}\left(0.886+4.64\alpha -13.32\alpha^{2}+14.72\alpha^{3}-5.6\alpha^{4}\right)$$

where α=a/W; a is the fatigue crack length, mm; Δ*P* is the applied loading amplitude, N; *B* is the specimen thickness, mm; and *W* is the specimen width, mm.

In Figure 3b it is obvious that FCGR almost linearly decreases with Δ*K* for BM specimen, whereas several inflection points can be observed in FCGRs for HAZ specimen. This is probably due to the non-uniform microstructure with much coarser grains in HAZ, as shown in Figure 2. At low Δ*K*, the HAZ shows much higher FCGRs than those of BM and WM, indicating the weakest fatigue crack growth resistance in the whole welded joint. Specifically, the FCGR of HAZ in this region is approximately two times higher than that of BM and WM. This is expected since the coarse-grained materials generally possess low fatigue strength. Deng et al. [13] reported that, in the dissimilar 9Cr-CrMoV welded joint, the 9Cr-HAZ and CrMoV-HAZ with narrow size were the weaker parts because of the lower FCG threshold compared to BM and WM. This is consistent with our results that HAZ possesses higher FCGR at low Δ*K* values. With further propagation of crack, the FCGR becomes comparable with that of BM.

On the other hand, the FCGR of WM reached as much as 1.5 times larger than that of BM in the intermediate region, which is probably due to the effect of tensile welding residual stress in WM specimen [31]. Previous work performed by our group measured the welding residual stress of the 2.25Cr1Mo0.25V welded joint by using the hole-drilling method, and the results showed that tensile residual stress was mainly distributed in the fusion zone [22]. Some studies have pointed out that the welding residual stress would partly retained in fracture mechanics specimens extracted from the welded joints [32,33]. Moreover, it has been reported that the FCGR of the WM can be significantly increased by the retained tensile residual stress [31], which is consistent with the present results. With further increase of Δ*K*, the slope of the FCGR of WM gradually decreases and finally the FCGR becomes comparable with that of BM.

The FCGR data of different specimens can be fitted using the following Paris–Erdogan crack growth law [34]:

(2) $$\frac{da}{dN}=C{\left(\Delta K\right)}^{m}$$

where *C* and m are material constant, which can be determined experimentally and are dependent on the test conditions. This formula can also be written as:

(3) $$log\left(\frac{da}{dN}\right)=m\log\left(\Delta K\right)+\log C$$

This equation means that the relationship between FCGR and Δ*K* is linear in the double logarithmic axes, as shown in Figure 3b. The Paris–Erdogan law coefficients and the corresponding correlation coefficient R^2^ are given in Table 3. The obtained values of R^2^ close to 1 show a good fit to the Paris–Erdogan law. It is obvious from Table 3 that the FCGR data of BM shows the best correlation with Δ*K*, while the HAZ shows the poorest. 

### 3.3. Fracture Morphology

**Figure 4:**
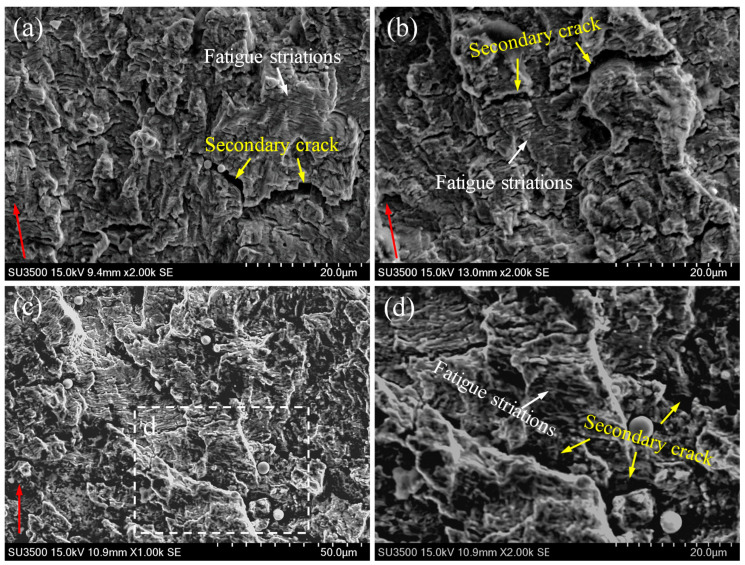
Fatigue fracture surfaces of (**a**) BM, (**b**) HAZ and (**c**,**d**) WM specimens at Δ*K* value of approximately 28.5 MPa·m^1/2^. Typically, the red arrows represent the crack growth direction, the white arrows represent the fatigue striations, and the yellow arrows indicate the secondary cracks.

**Figure 5:**
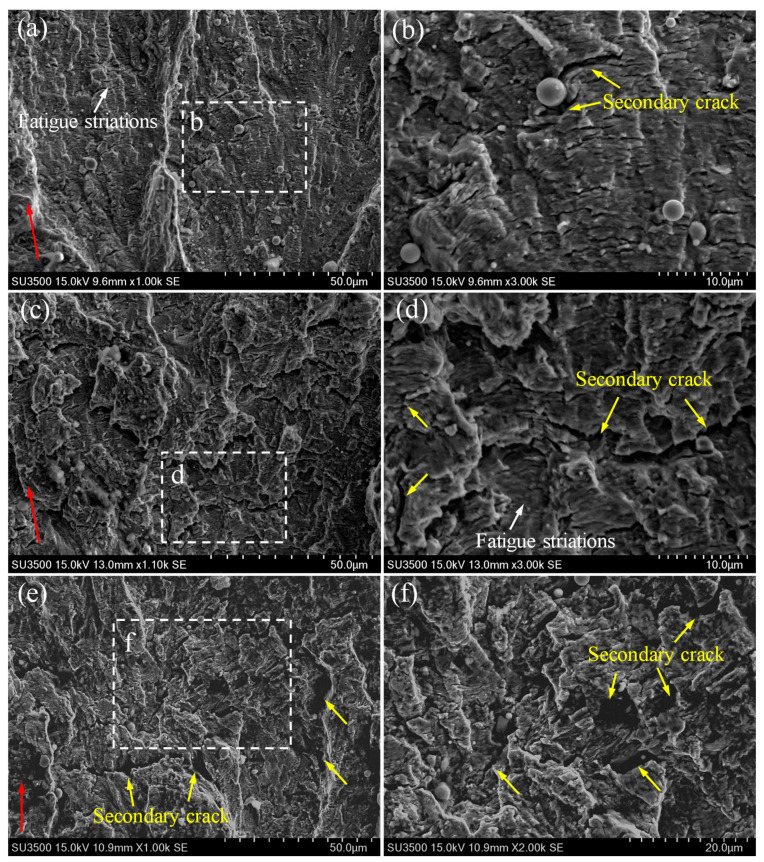
Fatigue fracture surfaces of (**a**,**b**) BM, (**c**,**d**) HAZ and (**e**,**f**) WM specimens at Δ*K* value of approximately 33 MPa·m^1/2^. Typically, the red arrows represent the crack growth direction, the white arrows represent the fatigue striations, and the yellow arrows indicate the secondary cracks. Some secondary cracks perpendicular to fatigue striations can be found in (**d**–**f**).

**Figure 6:**
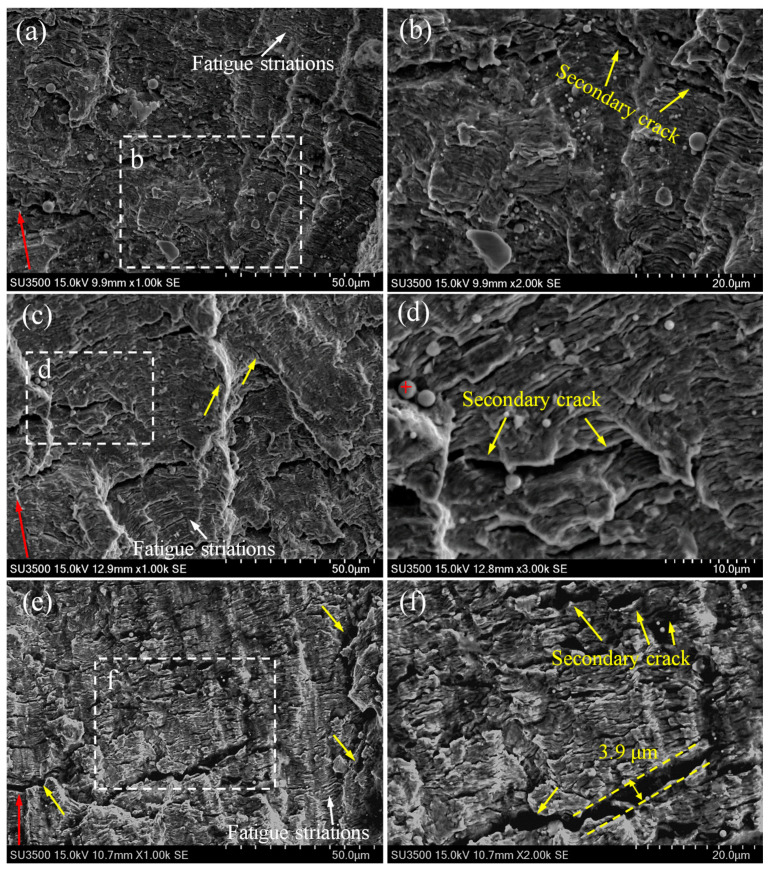
Fatigue fracture surfaces of (**a**,**b**) BM, (**c**,**d**) HAZ and (**e**,**f**) WM specimens at Δ*K* value of approximately 55 MPa·m^1/2^. Typically, the red arrows represent the crack growth direction, the white arrows represent the fatigue striations, and the yellow arrows indicate the secondary cracks. Some secondary cracks perpendicular to fatigue striations can be found in (**c**,**f**).

To investigate the fatigue mechanisms of different zones in the 2.25Cr1Mo0.25V steel welded joint, the fracture surfaces of the fatigued CT specimens were analyzed by using SEM. Typical fatigue fracture surfaces of BM, HAZ and WM at different stress intensity factor amplitudes Δ*K* (approximately 28.5, 33 and 55 MPa·m^1/2^) are displayed in Figure 4, Figure 5 and Figure 6. These figures obviously show transgranular fatigue fracture for all specimens. Fatigue striations, which are essentially a series of parallel and slightly curved stripes perpendicular to the crack growth direction, were frequently observed in the fracture surfaces of all specimens, as shown by the white arrows. The formation of fatigue striations can be explained by the crack-tip blunting mechanism proposed by Laird [35]. When the stress reaches the maximum compressive stress in a cycle, the crack tip is fully closed and sharpened. With the increase of the stress to maximum tensile stress, the crack tip reopens and becomes blunted. The crack tip becomes sharpened again when the stress changes to compressive stress. Thus, with the fatigue cycle going, the crack constantly propagates forward, leaving a series of striations on fracture surface. Moreover, the fatigue striation generally corresponds to the crack growth rate. It can be seen from these figures that the fatigue striation spacing becomes large and evident for each specimen with the increase of Δ*K* and FCGR. 

**Figure 7:**
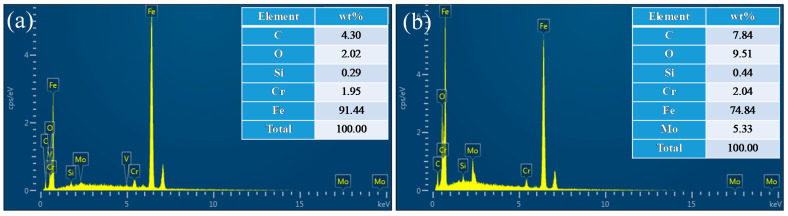
Energy dispersive spectrum (EDS) profiles of (**a**) the matrix, and (**b**) the inclusion marked by red “+” in Figure 6d.

Besides fatigue striations, numerous secondary cracks can be observed in all specimens, as shown by the yellow arrows in these figures. It is apparent that a large majority of the secondary cracks are perpendicular to the crack growth direction (i.e., parallel to fatigue striations). This secondary cracking phenomenon has been well explained by Gauthier et al. [36] and supported by many experimental observations and results [37]. During fatigue loading, tension stresses appear at the bottom of each striation, and they might be high enough to promote the transformation of some micro-striations into micro-cracks, consequently leading to a large amount of secondary cracks parallel to fatigue striations in the fracture surface [36]. On the other hand, several secondary cracks parallel to the crack growth direction (i.e., perpendicular to fatigue striations) are also observed, as shown in Figure 5d–f and Figure 6d,e by the yellow arrows. Additionally, fractographic examinations at a higher magnification reveal that inclusions are embedded in some secondary cracks, as displayed in Figure 5b and Figure 6d. Energy dispersive spectrum (EDS) analysis results (see Figure 7) further reveal that the chemical composition of inclusion contains higher contents of C and O than those in the matrix, indicating that the presence of inclusions assisted the initiation and growth of secondary cracks.

However, when carefully comparing the fracture surface features of BM, HAZ and WM specimens, it can be seen that the fracture surface of the WM specimen shows a significantly larger density of secondary cracks and micro-holes than those of BM and HAZ specimens. Specifically, some of the secondary cracks exhibit much larger size with significantly increased length and width. For instance, the fractograph of the fracture surface of WM at a higher Δ*K* value of 55 MPa·m^1/2^ exhibits a large secondary crack with the width up to 3.9 μm, as shown in Figure 6f. It has been reported that the secondary cracks would share a part of energy during crack extension process, decreasing the crack driving force and thus reducing the crack growth rate [38,39]. It can be; thus, inferred that the larger amount of secondary cracks with larger size are likely to consume more energy during crack growth, resulting in reduced FCGR and enhanced fatigue life. This is the reason why the slope of FCGR of WM gradually decreases at high Δ*K* values and finally becomes comparable to that of BM in this study.

In addition, it is worth noting that no evident difference in surface roughness can be seen from the SEM photographs of fracture surfaces of different specimens at the same Δ*K*. Previous investigations have reported that the grain size can significantly affect the crack growth path and FCGR in various materials [40,41,42]. In particular, severe crack deflection is prone to occur in coarse-grained materials, leading to rougher fatigue fracture surface than that in fine-grained material. The rough crack surface can further promote the roughness-induced crack closure (RICC) effect, reducing the effective driving force for crack propagation and; therefore, leading to a low FCGR [40,41,42]. In this study, despite the fact that BM exhibits fine equiaxed bainite grains and the microstructures of HAZ and WM consist of much coarser grains, their fatigue fracture surfaces show no significant difference in surface roughness. Therefore, the FCGRs of these specimens could be comparable in most of the fatigue life.

### 3.4. Acoustic Emission Analysis

**Figure 8:**
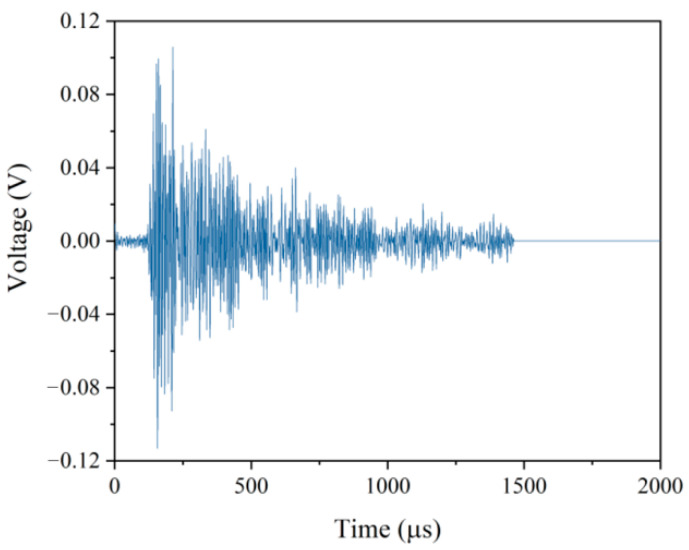
A typical acoustic emission (AE) waveform collected at fatigue loading time of 4284 s from FCG process of BM specimen. The energy and peak amplitude were calculated as 18 aJ and 61 dB, respectively.

In order to investigate the effect of microstructures on FCG behavior of the 2.25Cr1Mo0.25V steel welded joint, in this study, the AET was used to simultaneously monitor the AE signals generated during the FCG process, and a great number of AE waveforms were collected by using a high sensitivity AE sensor, as shown in Figure 1a. Figure 8 shows a typical complete AE waveform generated by crack growth of 2.25Cr1Mo0.25V steel. Numerous AE descriptors available to characterize the mechanical properties and microstructural changes can be extracted from each AE waveform. The commonly used descriptors include peak amplitude, count, energy, rise time, duration, entropy and so forth [28,43]. In particular, the energy represents the measured area of the waveform envelop, which can be calculated by integrating the transient voltage of the recorded acoustic event over the period of time. This descriptor has been regarded as the primary attribute of AE waveform for detecting critical damages and discriminating different damage modes because it is able to reflect the intensity and activity of AE source. Moreover, the energy is less dependent on the selected threshold, which is predetermined by researchers for de-nosing before collecting AE signals [16]. Therefore, in the present study, the energy was mainly used to characterize the FCG behaviors of different specimens. In addition, it has been reported that the count and peak amplitude are two important descriptors for characterizing the damage intensity during crack growth in various materials [16,18,21]. Specifically, the count is defined as the number of times within the duration, when the transient voltages exceed the threshold. While the peak amplitude represents the largest voltage peak in the recorded waveform. Thus, the count and peak amplitude were also calculated from the waveforms to provide supporting information for AE results.

**Figure 9:**
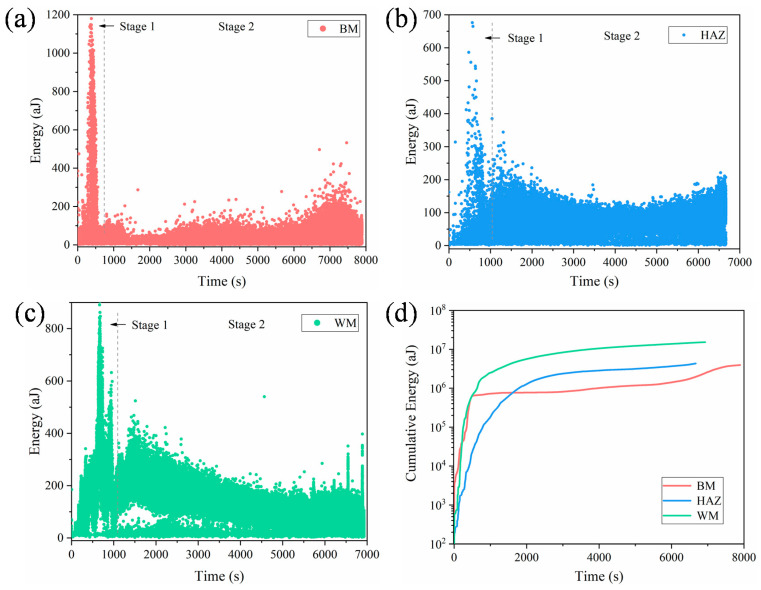
The AE energy versus fatigue loading time during the fatigue crack growth (FCG) test for (**a**) BM, (**b**) HAZ and (**c**) WM specimens. (**d**) Cumulative energy versus time of all specimens. Two obvious stages can be seen from the evolution of AE energy for all specimens.

The evolutions of energy with respect to fatigue loading time for different specimens were presented in Figure 9a–c. It is apparent from these figures that two damage stages can be easily discriminated. At the beginning of stage 1, signals with low energies occur due to extensive plastic deformation caused by stress concentration at the notch tip of CT specimens. Subsequently, a sudden increase of signals with high energies emerge, which is related to physical micro-crack initiation. The energy values in this stage are much higher than those in other fatigue loading time, and can be as high as 1200 aJ, as shown in Figure 9a for BM specimen. Several researchers have reported that the intensities of AE signals generated from crack initiation are much larger than those caused by plastic deformation and stable crack growth [16,44,45], which is consistent with our results. In stage 2, the variation of energy become constant and most of the energy values are below 200 aJ. This stage is reasonable related to stable crack growth stage, where the relationship between the FCGR and Δ*K* follows the Paris law. It is also worth noting that an increase of energy occurs again at the end of stage 2, which is due to the rapid crack growth at high Δ*K*. However, this sudden increase behavior is not very obvious as that caused by the crack initiation in stage 1. Based on above analyses, one can conclude that AE energy is successful in discriminating two damage stages during FCG process of the 2.25Cr1Mo0.25V steel welded joint. The first stage corresponds to crack initiation, and the second stage is related to stable and rapid crack growth.

**Figure 10:**
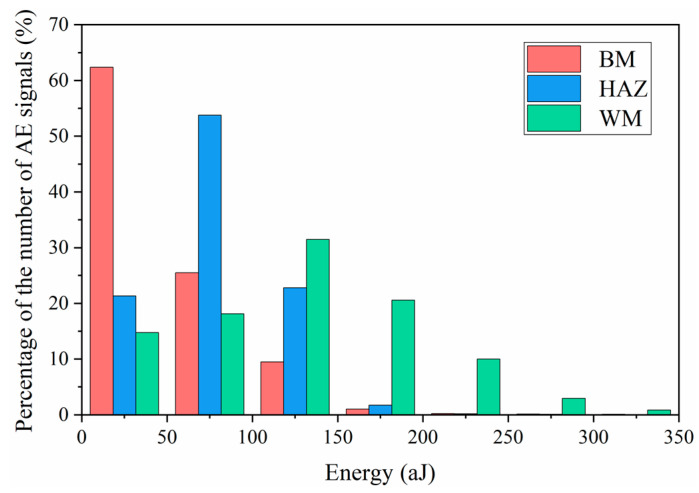
The statistical results of energy distribution of different specimens.

Figure 9d shows the evolution of cumulative energy with respect to time for different specimens. It is obvious that the WM specimen possesses the largest cumulative energy in most of the fatigue life, while the BM specimen shows the lowest energy value despite its longest fatigue life among the three specimens. To further investigate the difference in AE signals of the three specimens, the statistical calculation of energy distribution was carried out, and the result is shown in Figure 10. It can be seen that more than 70 % of AE energies for BM and HAZ specimens are below 100 aJ, while AE energies of WM specimen distributes in much greater values. In particular, more than 60 % of AE energies are greater than 100 aJ, leading to the largest value of cumulative energy for WM. This means that the FCG of the WM specimen is able to generate AE signals with high energy. It is important to note that the fracture surface of WM specimen shows a significantly larger density of secondary cracks with larger size, compared to those of BM and HAZ specimens. The initiation and propagation of secondary cracks would consume additional energy of crack tip during FCG, thus generating AE signals with high energy. Han et al. [21] reported that the inclusions in the weld specimen of a micro-alloyed steel promoted the formation of micro-voids and micro-cracks, resulting in higher AE counts rate in weld specimen, which is consistent with our results. Therefore, the significantly enhanced AE energy in the WM specimen is mainly attributed to the formation and propagation of numerous secondary cracks.

**Figure 11:**
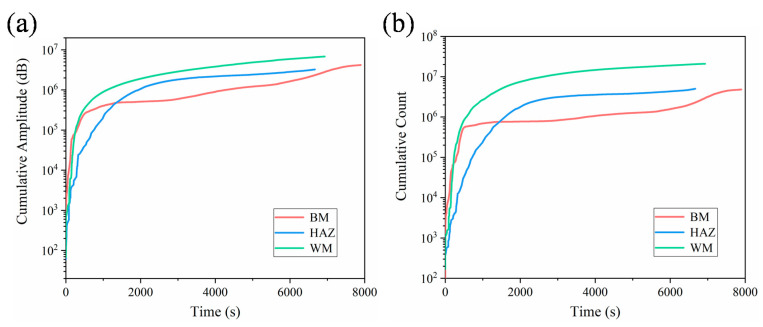
(**a**) The cumulative amplitude and (**b**) cumulative count with respect to fatigue loading time for different specimens.

It can be also obtained from Figure 10 that the most of the AE energies in the BM specimen distributes in lower values than those in HAZ and WM specimens. This indicates that the fine equiaxed microstructures in the BM specimen are prone to generate AE signals with low energy, while the coarse heterogeneous microstructures would generate signals with high energy. Previous studies [18,19,46] have reported that the coarse-grained microstructures in various metallic materials can cause an important increase in the activity and intensity of AE signals during FCG, which agrees with the present results. This is probably due to the fact that the finer grain size with larger area of grain boundaries would impede the dislocation motion, which leads to a decrease of AE energy in the BM specimen. Figure 11 shows the evolutions of cumulative amplitude and cumulative count for different specimens, presenting similar result with that of cumulative energy. This indicates that AE is able to successfully characterize microstructural changes during FCG by extracting proper descriptors from AE waveforms.

In brief, this study investigated the FCG behaviors and fatigue fracture mechanisms of different zones in the 2.25Cr1Mo0.25V steel welded joint by compact tension tests combined with AE monitoring. The coarse-grained WM specimen shows lower fatigue life but higher AE intensity than BM. The HAZ specimen also shows higher AE intensity than that of BM despite the fact that HAZ possesses the poorest fatigue life and similar fracture surface features with BM. Therefore, further studies need to be performed to comprehensively investigate the effect of microstructures, especially the grain size, on FCGR and the corresponding AE behaviors.

## 4. Conclusions

In this study, the fatigue crack growth behavior and fatigue fracture mechanism of the 2.25Cr1Mo0.25V steel welded joint were investigated. Fatigue tests were performed on CT specimens with notches in BM, HAZ and WM. Moreover, the AE signals generated from FCG processes were monitored simultaneously and investigated to gain insights into damage identification and microstructural effects of the 2.25Cr1Mo0.25V steel welded joint. Major conclusions of this study are stated as follows:

1. The microstructure of BM is fine granular bainite, while the WM shows coarser bainite grains. The HAZ exhibits the most significantly inhomogeneity, with large dispersion of grain size.
2. The FCGRs of HAZ specimen are approximately two times higher at low Δ*K* values than those of BM and WM, which could be attributed to non-uniform microstructure with much coarser grains. The BM shows the most superior fatigue resistance, which is due to the fine equiaxed bainite grains. The relationship between FCGR and Δ*K* follows the Paris law.
3. SEM analyses reveal the transgranular fracture with fatigue striations as the dominant fracture mechanism for all specimens. Moreover, the fracture surface of the WM specimen shows a significantly larger density of secondary cracks with large size compared to BM and HAZ specimens.
4. The FCG in the WM specimen generates more AE activity with higher energy values of AE signals compared to BM and HAZ specimens. This is attributed to the combined influence of the formation of numerous secondary cracks and coarse-grained microstructures.

## Figures and Tables

**Table 1:** Mechanical properties of 2.25Cr1Mo0.25V steel.

Yield Strength (MPa)	Ultimate Strength (MPa)	Percentage of Elongation (%)	Percentage of Area Reduction (%)
569	678	29	81

**Table 2:** Chemical composition of base metal (BM) and weld metal (WM) (wt.%).

Element	C	Si	Mn	P	S	Cr	Mo	V	Al	Ni	Cu
BM	0.15	0.10	0.54	0.009	0.01	2.30	0.98	0.30	0.05	-	-
WM	0.12	0.22	1.07	0.004	0.004	2.45	1.03	0.42	-	0.03	0.11

**Table 3:** Paris–Erdogan law parameters for different specimens.

Specimen	C	m	R^2^
BM	6.309^−10^	3.454	0.986
HAZ	8.433^−9^	2.772	0.899
WM	2.564^−10^	3.746	0.950

## Data Availability

The data used to support the findings of this study are available from the corresponding author upon request.
